# The Medial Amygdala-Medullary PrRP-Synthesizing Neuron Pathway Mediates Neuroendocrine Responses to Contextual Conditioned Fear in Male Rodents

**DOI:** 10.1210/en.2013-1411

**Published:** 2014-05-30

**Authors:** Masahide Yoshida, Yuki Takayanagi, Tatsushi Onaka

**Affiliations:** Division of Brain and Neurophysiology, Department of Physiology, Jichi Medical University, Shimotsuke-shi, Tochigi-ken 329–0498, Japan

## Abstract

Fear responses play evolutionarily beneficial roles, although excessive fear memory can induce inappropriate fear expression observed in posttraumatic stress disorder, panic disorder, and phobia. To understand the neural machineries that underlie these disorders, it is important to clarify the neural pathways of fear responses. Contextual conditioned fear induces freezing behavior and neuroendocrine responses. Considerable evidence indicates that the central amygdala plays an essential role in expression of freezing behavior after contextual conditioned fear. On the other hand, mechanisms of neuroendocrine responses remain to be clarified. The medial amygdala (MeA), which is activated after contextual conditioned fear, was lesioned bilaterally by infusion of *N*-methyl-d-aspartate after training of fear conditioning. Plasma oxytocin, ACTH, and prolactin concentrations were significantly increased after contextual conditioned fear in sham-lesioned rats. In MeA-lesioned rats, these neuroendocrine responses but not freezing behavior were significantly impaired compared with those in sham-lesioned rats. In contrast, the magnitudes of neuroendocrine responses after exposure to novel environmental stimuli were not significantly different in MeA-lesioned rats and sham-lesioned rats. Contextual conditioned fear activated prolactin-releasing peptide (PrRP)-synthesizing neurons in the medulla oblongata. In MeA-lesioned rats, the percentage of PrRP-synthesizing neurons activated after contextual conditioned fear was significantly decreased. Furthermore, neuroendocrine responses after contextual conditioned fear disappeared in PrRP-deficient mice. Our findings suggest that the MeA-medullary PrRP-synthesizing neuron pathway plays an important role in neuroendocrine responses to contextual conditioned fear.

Fear is an evolutionarily conserved emotion, which initiates a series of defensive machineries for adaptation to threatening events that is crucial for survival. The amygdala is a well-defined subcortical nuclear group and a principal component of the neural circuitry of innate and learned fear (1, 2). The memory of learned fear has been quantitatively characterized by a conditioned fear paradigm. During fear conditioning, an initially neutral conditioned stimulus acquires biological significance by coupling with an aversive stimulus. After learning this association, an animal responds to the previously neutral conditioned stimulus with a series of defensive reactions, which include freezing behavior and neuroendocrine responses (3).

Contextual conditioned fear facilitates release of oxytocin, ACTH, and prolactin from the pituitary into systemic circulation (4). It has been suggested that these neuroendocrine responses influence cardiovascular, metabolic, immune, and reproductive systems (5–8). Medullary noradrenergic neurons play an important role in neuroendocrine responses after contextual conditioned fear (9). Contextual conditioned fear activates noradrenergic neurons in the medulla oblongata and induces neuroendocrine responses. The neuroendocrine responses are blocked by disruption of noradrenergic fibers (10) or by administration of an α1-adrenoceptor antagonist (11). The medullary noradrenergic neurons send projections to the hypothalamus, and selective destruction of noradrenergic fibers projecting to the supraoptic nucleus (SON) impairs activation of oxytocin-synthesizing neurons in the SON after contextual conditioned fear (12).

A subset of noradrenergic neurons in the caudal nucleus tractus solitarii (NTS) and ventrolateral medulla (VLM) contains prolactin-releasing peptide (PrRP), and PrRP coexists exclusively in the noradrenergic neurons (13–15). PrRP is involved in neuroendocrine responses after contextual conditioned fear. Contextual conditioned fear activates medullary PrRP-synthesizing neurons projecting to the hypothalamus, and most activated neurons in the NTS show expression of PrRP. Immunoneutralization of endogenous PrRP with intracerebroventricular monoclonal anti-PrRP antibodies impairs oxytocin secretion after contextual conditioned fear (16).

Lesion and anatomical tracing studies have demonstrated the importance of the amygdala for expression of fear conditioning responses. Information concerning a contextual conditioned stimulus and an aversive stimulus converges during contextual fear conditioning in the basolateral amygdala. The signal from the basolateral amygdala is conveyed to other amygdaloid nuclei including the central amygdala, which is the major source of extra-amygdaloid outputs. The central amygdala sends projections to the brainstem, which controls behavioral expression of fear (freezing behavior) (3, 17). Selective lesions of the central amygdala after learning block expression of freezing behavior (18) but not release of corticosterone or prolactin (19) in response to contextual conditioned fear. Thus, output nuclei of the amygdala controlling neuroendocrine fear responses remain to be clarified. In the amygdala, expression of Fos protein in response to contextual conditioned fear occurs in the medial amygdala (MeA) (20). However, the function of the MeA in the control of contextual conditioned fear responses is unknown. To test the hypothesis that the MeA is essential for neuroendocrine responses to contextual conditioned fear, we investigated whether excitotoxic lesions of the MeA change neuroendocrine responses. We found that the MeA is important for neuroendocrine responses to contextual conditioned fear and activation of medullary PrRP-synthesizing neurons. In addition, we examined contextual conditioned fear responses in PrRP-deficient mice and found that PrRP plays an essential role in neuroendocrine responses after contextual conditioned fear.

## Materials and Methods

### Animals

Male rats (9–11 weeks old, Slc: Wistar, Japan SLC) and male mice (14–15 weeks old, PrRP-deficient and wild-type mice on a C57BL/6N background) (21) were housed under a 12-hour light, 12-hour dark cycle (lights on 7:30 am) at 22°C ± 2°C and 55% ± 15% relative humidity. Food and water were available ad libitum. Animal experiments were carried out after receiving approval from the Animal Experiment Committee of Jichi Medical University and were in accordance with the Institutional Regulations for Animal Experiments and Fundamental Guidelines for Proper Conduct of Animal Experiments and Related Activities in Academic Research Institutions under the jurisdiction of the Ministry of Education, Culture, Sports, Science, and Technology.

### MeA lesions

Rats were administered atropine (0.4 mg/kg, ip) to suppress respiratory secretion and then anesthetized with pentobarbital (50 mg/kg, ip) and placed in a stereotaxic frame. Lesion coordinates (2.7 mm caudal to the bregma, ± 3.2 mm lateral to the midline, and 9.0 mm below the skull) were based on the rat brain atlas (22). An excitotoxin, *N-*methyl-d-aspartate (NMDA) (20 μg/μL dissolved in 0.1M phosphate buffer, pH 7.4) or its vehicle was infused through a cannula (diameter, 0.1 mm). Infusion was carried out at a rate of 0.05 μL/min using a microinfusion pump (Narishige; MO-81). NMDA (4 μg in 0.2 μL per side) or the vehicle was injected bilaterally. The tip of the infusion cannula and lesion sites were examined after Nissl staining and immunocytochemical detection of NeuN. Lesion sites were evaluated on the basis of the extent of neuronal cell loss. Lesion area was traced by using CellSens Dimension software (Olympus), and lesion size was measured in 7 sections per rat.

### Contextual fear conditioning in rats and mice

Contextual fear conditioning was conducted (23–25) in a similar way to that described previously with minor modifications (16). Rats were kept in a room next to the room for contextual fear conditioning. Rats received a habituation session twice a day on the first and second days. In the habituation session, rats were transferred to an experimental room, placed in an experimental box (30 × 30 × 60 cm) equipped with a grid floor made of stainless steel tubes, and left there for 10 minutes. The same box was used throughout the experiments. On the third day, rats received a training session. In the training session, rats in the shocked group were placed in the box for 5 minutes and then given electric shocks (0.8 mA, 50 Hz, 1 second) repeated every 30 seconds for 5 minutes. The nonshocked control rats received the same habituation session and training procedures without electric shocks. On the fourth day, NMDA or the vehicle was infused into the MeA as described above. Seven days after the infusion, rats received a testing session. In the testing session, rats were placed in the box where they had received shocks at the time of training and were left there for 4 minutes. Trunk blood was collected by decapitation immediately after the testing stimuli. For immunohistochemical detection, the rats were returned to their home cages after completion of testing stimuli. At 110 minutes after the initiation of the testing session, the rats were anesthetized with sodium pentobarbital and perfused transcardially with heparinized saline (20 U/mL) followed by 4% paraformaldehyde in 0.1M phosphate buffer (pH 7.4) for 15 minutes. The brains were immediately removed and postfixed in 4% paraformaldehyde overnight and then placed in 30% sucrose in 0.1M phosphate buffer until they sank. The brains were frozen on dry ice and stored at −80°C until immunocytochemical examination.

In experiments with mice, mice were kept in a room next to the room for contextual fear conditioning. Mice received a training session on the first day and a testing procedure on the second day. In the training session, mice in the shocked group were placed in an experimental box (20 × 20 × 20 cm) equipped with a grid floor made of stainless steel tubes, left there for 2 minutes, and then given electric shocks (0.5 mA, 50 Hz, 1 second) repeated every 30 seconds for 5 minutes. The nonshocked control mice received the same training procedures without electric shocks. The same box was used throughout the experiments. In the testing session, mice were placed in the box where the mice had received shocks at the time of training and were left there for 5 minutes. Trunk blood was collected by decapitation immediately after the testing stimuli.

Cumulative time periods in which the rats and mice exhibited freezing behavior and total distance of rats during the testing session were measured by Image FZ (O'Hara & Co, Japan), which was produced on the basis of the public domain NIH Image program (developed at the US National Institutes of Health and available on the Internet at http://rsb.info.nih.gov/nih-image/).

### Novel environmental stimuli in rats

Rats were injected with NMDA or the vehicle into the MeA as described above. On the seventh day after infusion of NMDA or the vehicle, the rats were placed in a white-painted plastic pail (39 cm diameter, 60 cm height) for 3 minutes. Trunk blood was collected immediately after the testing. Rats in the control group were kept in their home cages until collection of trunk blood.

### Measurement of hormone concentrations

Plasma concentrations of oxytocin, ACTH, and prolactin were determined by RIA with specific antioxytocin, anti-ACTH, and antiprolactin antisera, respectively, as described previously (4, 16). Coefficients of interassay variations were 4% for oxytocin, 5% for ACTH, and 7% for prolactin. Intra-assay variations were 10% for oxytocin, 10% for ACTH, and 10% for prolactin. The minimum detection limits were 2 pg/mL for oxytocin, 10 pg/mL for ACTH, and 8 ng/mL for prolactin.

### Immunohistochemical detection of NeuN

Coronal sections of rat brains were cut at 30 μm with a freezing sledge microtome. Every fourth section of the amygdala was collected and processed for immunohistochemical detection of NeuN. The sections were incubated for 15 minutes with 1.5% H_2_O_2_ solution to block endogenous peroxidase, with 10% normal goat serum for 1 hour, and then with a biotinylated mouse monoclonal antibody against NeuN (diluted 1:2000; Millipore) for 48 hours at 4°C, followed by incubation with avidin-biotinylated horseradish peroxidase complex (Vector Laboratories) for 60 minutes at room temperature. NeuN immunoreactivity was visualized as a brown cytoplasmic precipitate using the 3,3′-diaminobenzidine procedure. Seven sections were examined for the amygdala in each rat.

### Immunohistochemical detection of Fos protein and PrRP

Coronal sections of rat brains were cut at 30 μm with a freezing sledge microtome. Every fourth section of the medulla oblongata was collected and processed for immunohistochemical detection of Fos protein and PrRP, as described previously (16). In brief, sections were pretreated with 1% sodium borohydride for 20 minutes. The sections were incubated for 15 minutes with 1.5% H_2_O_2_ solution to block endogenous peroxidase, with 10% normal goat serum for 1 hour, and then with a rabbit polyclonal antibody against Fos protein (diluted 1:10 000; Oncogene Science) for 48 hours at 4°C, followed by incubation with goat antirabbit IgG-peroxidase complex (diluted 1:1000; Vector Laboratories) for 24 hours at 4°C. Fos protein immunoreactivity was visualized as a black nuclear precipitate using a glucose oxidase-based, nickel-intensified, 3,3′-diaminobenzidine procedure. The sections were treated with 10% normal horse serum and then incubated with a mouse anti-PrRP antibody (5 μg/mL; Takeda Pharmaceutical Co) for 48 hours at 4°C, followed by incubation with biotinylated horse antimouse IgG (diluted 1:500; Vector Laboratories) for 120 minutes and then with avidin-biotinylated horseradish peroxidase complex (Vector Laboratories) for 30 minutes at room temperature. PrRP immunoreactivity was visualized as a brown cytoplasmic precipitate using the 3,3′-diaminobenzidine procedure. Seventeen sections were examined for the medullary NTS and VLM in each rat.

### Specific experimental procedures

#### Experiment 1: effect of MeA lesion on contextual conditioned fear

Rats received habituation and training sessions, as described above. On the day after the training session, NMDA or the vehicle was infused into the MeA. Seven days after the infusion, rats received a testing session. Freezing behavior during the testing stimuli was observed, and plasma hormone concentrations immediately after testing stimuli were measured. The number of rats in each group was 6 to 8.

#### Experiment 2: effect of MeA lesion on novel environmental stimuli

Rats were injected with NMDA or the vehicle into the MeA, as described above. Seven days after the surgical operation for MeA lesions, rats were exposed to novel environmental stimuli. Plasma hormone concentrations immediately after testing stimuli were measured. The number of rats in each group was 8 or 9.

#### Experiment 3: effect of MeA lesion on activation of medullary PrRP-synthesizing neurons after contextual conditioned fear

Rats received habituation and training sessions, as described above. The day after the training session, NMDA or the vehicle was infused into the MeA. Seven days after the infusion, rats received a testing session. After completion of testing stimuli, the rats were returned to their home cages. At 110 minutes after initiation of the testing session, brains were obtained and processed for immunohistochemistry, as described above. The number of rats in each group was 9 to 12.

#### Experiment 4: effects of PrRP gene deficiency on contextual conditioned fear

Mice received contextual conditioned fear, as described above. Freezing behavior during testing stimuli was observed, and plasma hormone concentrations immediately after testing stimuli were measured. The number of mice in each group was 8 to 10.

### Statistics

Data are expressed as means ± SEM. Data were analyzed by two-way ANOVA followed by Fisher's Protected Least Significant Difference multiple-comparison test, Spearman correlation, and repeated-measures two-way ANOVA. *P* < .05 was considered statistically significant.

## Results

### Impairment of neuroendocrine responses but not freezing behavior after contextual conditioned fear in MeA-lesioned rats

**Figure 1.**
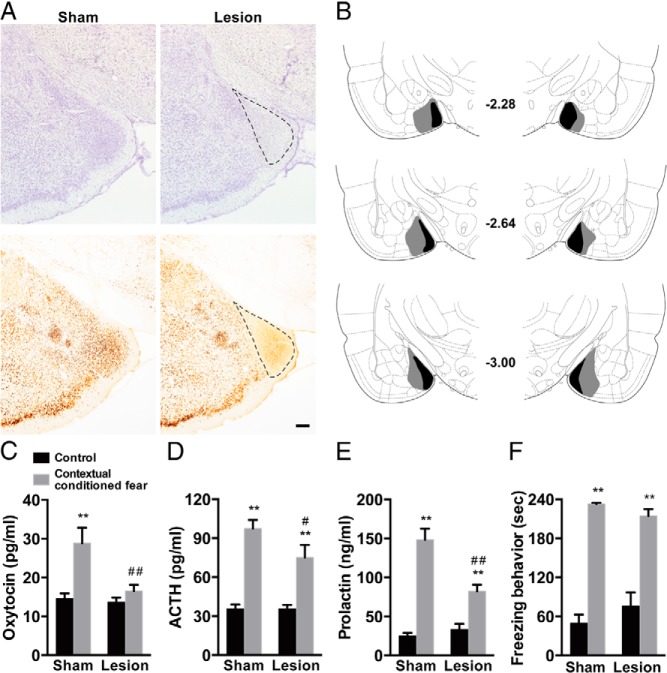
Neuroendocrine responses and freezing behavior after contextual conditioned fear in sham-lesioned and MeA-lesioned rats. A, Representative photomicrographs showing sham lesion and MeA lesion sites are presented. Lesion sites were verified using Nissl staining (top panels) and NeuN immunoreactivity (bottom panels). B, Largest (gray) and smallest (black) lesion sites in rostral to caudal MeA coronal levels. Numbers indicate millimeters posterior from bregma. C–F, Plasma concentrations of oxytocin (C), ACTH (D) and prolactin (E), and freezing behavior (F) after control or contextual conditioned fear in sham-lesioned and MeA-lesioned rats. The MeA lesion significantly impaired neuroendocrine responses to contextual conditioned fear. Broken lines indicate lesioned areas. Scale bar, 200 μm. **, *P* < .01 compared with control rats; ##, *P* < .01; #, *P* < .05 compared with corresponding groups of sham-lesioned rats; n = 6–8.

We first investigated whether MeA lesions impair the expression of neuroendocrine and behavioral responses after contextual conditioned fear. An NMDA solution was infused into the MeA for lesioning. The lesion site characterized by loss of neurons as revealed by Nissl staining, and NeuN immunoreactivity was found immediately lateral to the optic tract and localized within the MeA. The basolateral amygdala and central amygdala were spared (Figure 1, A and B).

Plasma concentrations of oxytocin, ACTH, and prolactin were significantly increased after contextual conditioned fear in sham-lesioned rats (oxytocin, *P* < .01; ACTH, *P* < .01; prolactin, *P* < .01, multiple-comparison test). In the rats whose MeA was lesioned, contextual conditioned fear responses of oxytocin, ACTH, and prolactin were significantly impaired compared with those in sham-lesioned rats (oxytocin, *P* < .01; ACTH, *P* < .05; prolactin, *P* < .01, multiple-comparison test). Two-way ANOVA with oxytocin, ACTH, and prolactin as dependent variables demonstrated significant effects for lesion (*F*_1,26_ = 6.22, *P* < .05 for oxytocin; *F*_1,26_ = 7.62, *P* < .05 for prolactin), stimulus (*F*_1,26_ = 14.72, *P* < .001 for oxytocin; *F*_1,26_ = 80.66, *P* < .0001 for ACTH; *F*_1,26_ = 92.05, *P* < .0001 for prolactin), and interaction (*F*_1,26_ = 5.31, *P* < .05 for oxytocin; *F*_1,26_ = 15.29, *P* < .001 for prolactin), whereas the effects for lesion and lesion by stimulus interaction for ACTH showed only a trend toward significance (*F*_1,26_ = 3.10, *P* = .089 [lesion], and *F*_1,26_ = 3.59, *P* = .069 [interaction]) (Figure 1, C–E).

Freezing behavior was observed during the contextual fear stimuli in sham-lesioned rats (*P* < .01, multiple-comparison test) and was not impaired after MeA lesioning. Two-way ANOVA with freezing behavior as dependent variable demonstrated significant effects for stimulus (*F*_1,26_ = 133.59, *P* < .0001) but neither for lesion nor for lesion by stimulus interaction (*F*_1,26_ = 0.17, *P* = .68 [lesion], and *F*_1,26_ = 2.49, *P* = .13 [interaction]) (Figure 1F). These results suggest that the MeA is essential for full expression of neuroendocrine responses to contextual conditioned fear.

### Neuroendocrine responses to novel environmental stimuli in MeA-lesioned rats

**Figure 2.**
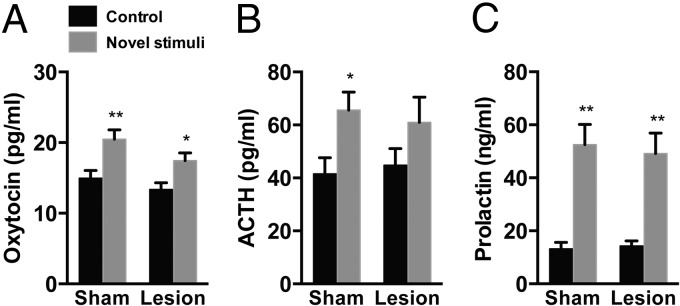
Neuroendocrine responses to novel environmental stimuli in sham-lesioned and MeA-lesioned rats. A–C, Plasma concentrations of oxytocin (A), ACTH (B), and prolactin (C) after novel environmental stimuli in sham-lesioned and MeA-lesioned rats. The MeA lesion did not significantly affect neuroendocrine responses to novel environmental stimuli. **, *P* < .01; *, *P* < .05 compared with control rats; n = 8 or 9.

We then examined the effects of MeA lesions on neuroendocrine responses to novel environmental stimuli. Plasma concentrations of oxytocin, ACTH, and prolactin were significantly increased after exposure to novel environmental stimuli in sham-lesioned rats (oxytocin, *P* < .01; ACTH, *P* < .05; prolactin, *P* < .01, multiple-comparison test). In the rats whose MeA was lesioned, these neuroendocrine responses were induced after novel environmental stimuli, although ACTH responses were not statistically significant (oxytocin, *P* < .01; prolactin, *P* < .01, multiple-comparison test). Plasma concentrations of these hormones after novel environmental stimuli were not significantly different in MeA-lesioned and sham-lesioned rats. Two-way ANOVA with oxytocin, ACTH, and prolactin as dependent variables demonstrated significant effects for stimulus (*F*_1,29_ = 17.21, *P* < .001 for oxytocin; *F*_1,29_ = 7.06, *P* < .05 for ACTH; and *F*_1,29_ = 40.77, *P* < .0001 for prolactin) but neither for lesion (*F*_1,29_ = 4.20, *P* = .05 for oxytocin; *F*_1,29_ = 0.01, *P* = .91 for ACTH; and *F*_1,29_ = 0.04, *P* = .84 for prolactin) nor for lesion by stimulus interaction (*F*_1,29_ = 0.38, *P* = .54 for oxytocin; *F*_1,29_ = 0.28, *P* = .60 for ACTH; and *F*_1,29_ = 0.15, *P* = .70 for prolactin) (Figure 2). These results are consistent with the view that the MeA is not essential for expression of neuroendocrine responses to novel environmental stimuli.

### Impaired Fos protein expression in medullary PrRP-synthesizing neurons after contextual fear stimuli in MeA-lesioned rats

**Figure 3.**
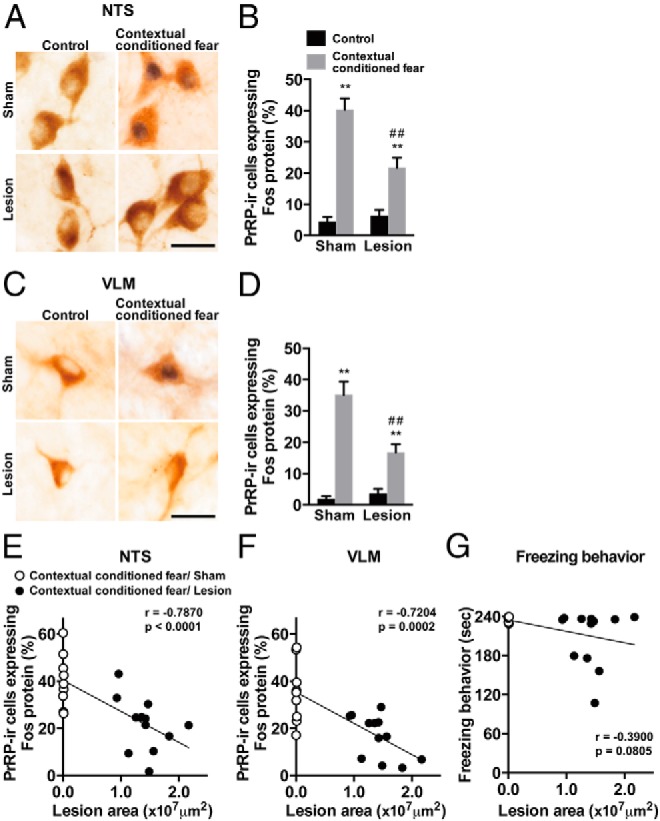
Activation of medullary PrRP-synthesizing neurons after contextual conditioned fear in sham-lesioned and MeA-lesioned rats. A and C, Photomicrographs showing Fos protein and PrRP immunoreactivity in the NTS (A) and VLM (C). B and D, Percentages of PrRP-immunoreactive neurons expressing Fos protein in the NTS (B) and VLM (D). Activation of PrRP-synthesizing neurons in the NTS and VLM after contextual conditioned fear was significantly impaired in MeA-lesioned rats. E–G, Correlations between size of MeA lesion area and the percentages of PrRP-immunoreactive neurons expressing Fos protein in the NTS (E) and VLM (F) or freezing behavior (G). Activation of PrRP-synthesizing neurons in the NTS and VLM negatively correlate with the size of MeA lesion area, whereas freezing behavior does not show a significant correlation. Brown cytoplasmic reactions indicate PrRP immunoreactivity and black nuclear reactions indicate Fos immunoreactivity. Scale bars, 20 μm. **, *P* < .01 compared with control rats; ##, *P* < .01 compared with corresponding groups of sham-lesioned rats; n = 9–12.

Contextual fear stimuli have been shown to activate PrRP-synthesizing neurons in the medulla oblongata that project to the hypothalamus (16). We thus examined effects of MeA lesions on activation of PrRP-synthesizing neurons in response to contextual conditioned fear by investigating the expression of Fos protein. The percentage of PrRP-synthesizing neurons expressing Fos protein in the NTS or VLM was significantly increased after contextual conditioned fear in sham-lesioned rats (NTS, *P* < .01; VLM, *P* < .01, multiple-comparison test). In the rats whose MeA was lesioned, the percentage of PrRP-synthesizing neurons expressing Fos protein was significantly lower than that in the sham-lesioned rats (NTS, *P* < .01; VLM, *P* < .01, multiple-comparison test). Two-way ANOVA with the percentage of PrRP-synthesizing neurons expressing Fos protein as the dependent variable demonstrated significant effects for NTS (*F*_1,38_ = 6.45, *P* < .05 [lesion]; *F*_1,38_ = 80.13, *P* < .0001 [stimulus]; *F*_1,38_ = 13.55, *P* < .001 [interaction]) and VLM (*F*_1,38_ = 7.86, *P* < .01 [lesion]; *F*_1,38_ = 75.95, *P* < .0001 [stimulus], *F*_1,38_ = 15.20, *P* < .001 [interaction]) (Figure 3, A–D). Expression of Fos protein in PrRP-synthesizing neurons in the NTS and VLM negatively correlated with size of MeA lesion areas (NTS, *r* = −0.7870, *P* < .0001; VLM, *r* = −0.7204, *P* = .0002). Freezing behavior did not significantly correlate with the size (*r* = −0.3900, *P* = .0805) (Figure 3, E–G). The total numbers of PrRP-synthesizing neurons in the NTS were 204.6 ± 9.5, 194.5 ± 10.4, 202.0 ± 11.7, and 208.6 ± 14.2 for rats subjected to sham-control, sham-contextual conditioned fear, lesion-control, and lesion-contextual conditioned fear, respectively. There were no significant differences among the groups. Two-way ANOVA with PrRP-synthesizing neurons as the dependent variable did not demonstrate significant effects for lesion (*F*_1,38_ = 0.03, *P* = .87), stimulus (*F*_1,38_ = 0.31, *P* = .58), or interaction (*F*_1,38_ = 0.42, *P* = .52). The total numbers of PrRP-synthesizing neurons in the VLM were 68.1 ± 4.3, 67.9 ± 5.2, 71.8 ± 5.3, and 67.9 ± 4.7 for rats subjected to sham-control, sham-contextual conditioned fear, lesion-control, and lesion-contextual conditioned fear, respectively. There were no significant differences among the groups. Two-way ANOVA with PrRP-synthesizing neurons as the dependent variable demonstrated no significant effects for lesion (*F*_1,38_ = 0.15, *P* = .70), stimulus (*F*_1,38_ = 0.11, *P* = .74), and interaction (*F*_1,38_ = 0.14, *P* = .71). Freezing behavior was not significantly influenced by MeA lesions (51.0 ± 12.2, 56.3 ± 8.7, 236.5 ± 1.4, and 208.3 ± 12.5 seconds for rats in the sham-control, lesion-control, sham-contextual conditioned fear, and lesion-contextual conditioned fear groups, respectively). Two-way ANOVA with freezing behavior as the dependent variable demonstrated significant effects for stimulus (*F*_1,38_ = 261.09, *P* < .0001) but neither for lesion nor for lesion by stimulus interaction (*F*_1,38_ = 0.10, *P* = .76 [lesion]; *F*_1,38_ = 2.59, *P* = .12 [interaction]). Total distances during the testing session were 261.0 ± 40.3, 253.2 ± 28.4, 56.7 ± 14.5, and 95.9 ± 17.1 cm for the rats in sham-control, lesion-control, sham-contextual conditioned fear, and lesion-contextual conditioned fear groups, respectively. Total distance was significantly greater in the control groups than in the conditioned fear groups, and MeA lesions had no significant effects. Two-way ANOVA with total distance as the dependent variable demonstrated significant effects for stimulus (*F*_1,38_ = 43.97, *P* < .0001) but neither for lesion nor for lesion by stimulus interaction (*F*_1,38_ = 0.07, *P* = .80 [lesion]; *F*_1,38_ = 0.76, *P* = .39 [interaction]). These results suggest that the MeA is essential for full activation of medullary PrRP-synthesizing neurons in response to contextual conditioned fear.

### Impaired neuroendocrine responses and enhanced freezing behavior after contextual fear stimuli in PrRP-deficient mice

**Figure 4.**
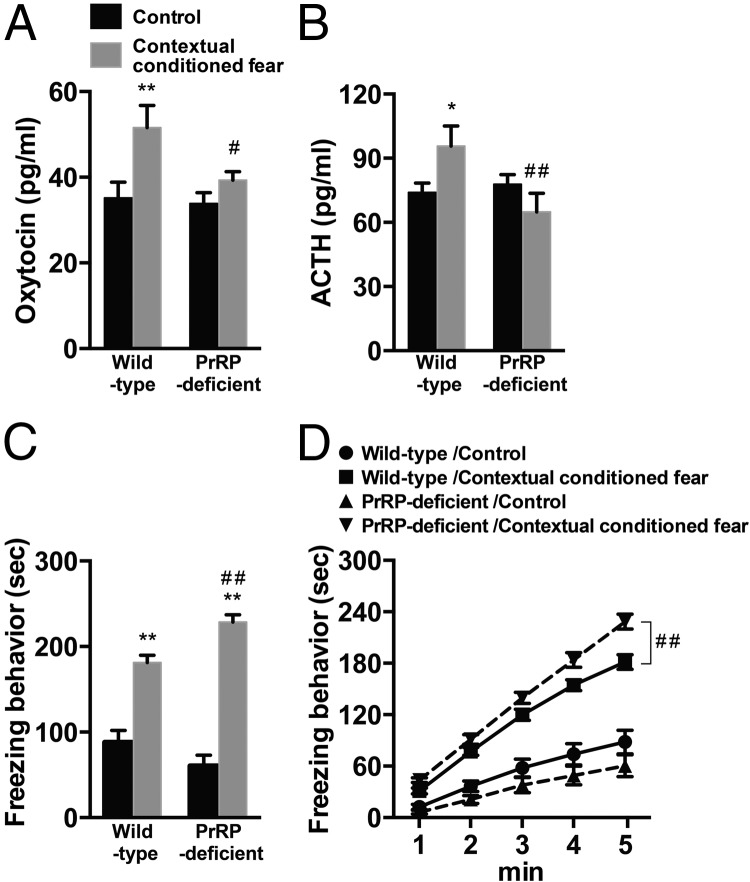
Neuroendocrine responses and freezing behavior after contextual conditioned fear in wild-type and PrRP-deficient mice. A–D, Plasma concentrations of oxytocin (A), ACTH (B), total time spent for freezing behavior during stimuli (C), and cumulative time spent for freezing behavior during time course of stimuli (D) after control or contextual conditioned fear in wild-type and PrRP-deficient mice. Neuroendocrine responses after contextual conditioned fear disappeared, whereas freezing behavior was augmented in PrRP-deficient mice. **, *P* < .01; *, *P* < .05 compared with control mice; ##, *P* < .01; #, *P* < .05 compared with corresponding groups of wild-type mice; n = 8–10.

We then investigated whether neuroendocrine responses after contextual conditioned fear were impaired in PrRP-deficient mice. Plasma concentrations of oxytocin and ACTH were significantly increased after contextual conditioned fear in wild-type mice (oxytocin, *P* < .01; ACTH, *P* < .05, multiple-comparison test). In PrRP-deficient mice, contextual conditioned fear responses of oxytocin and ACTH disappeared and plasma hormone concentrations after contextual conditioned fear were significantly lower than those in wild-type mice (oxytocin, *P* < .05; ACTH, *P* < .01, multiple-comparison test). Results of two-way ANOVA with oxytocin and ACTH as dependent variables were *F*_1,35_ = 8.92, *P* < .01 (stimuli); *F*_1,35_ = 4.01, *P* = .05 (genotype); *F*_1,35_ = 2.53, *P* = .12 (interaction) for oxytocin; and *F*_1,35_ = 0.35, *P* = .56 (stimulus); *F*_1,35_ = 3.68, *P* = .063 (genotype); *F*_1,35_ = 5.66, *P* < .05 (interaction) for ACTH (Figure 4, A and B). On the other hand, total time spent for freezing behavior during stimuli was significantly increased both in wild-type and PrRP-deficient mice (*P* < .01, multiple-comparison test). The magnitude of freezing behavior was larger in PrRP-deficient mice than in wild-type mice (*P* < .01, multiple-comparison test). Results of two-way ANOVA with total time spent for freezing behavior during stimuli as the dependent variable were *F*_1,35_ = 141.96, *P* < .0001 (stimulus); *F*_1,35_ = 0.94, *P* = .34 (genotype); and *F*_1,35_ = 11.51, *P* < .01 (interaction) (Figure 4C). Results of repeated-measures two-way ANOVA also indicated that the cumulative time spent for freezing behavior during the time course of the stimuli (1–5 minutes) was significantly longer in contextual conditioned PrRP-deficient mice than in contextual conditioned wild-type mice (*F*_1,72_ = 9.44, *P* < .01 [genotype]; *F*_1,72_ = 761.67, *P* < .0001 [time]; and *F*_1,72_ = 8.76, *P* < .0001 [interaction]) (Figure 4D). These results suggest that PrRP is crucial for neuroendocrine responses to contextual conditioned fear.

## Discussion

Contextual conditioned fear or exposure to a novel environment facilitates release of oxytocin, ACTH, and prolactin. In the present study, neuroendocrine responses to contextual conditioned fear, but not those to novel environmental stimuli, were impaired after MeA lesioning, suggesting involvement of the MeA in neuroendocrine responses to contextual conditioned fear. Some of the medullary PrRP-synthesizing neurons directly project to the hypothalamus (16). Oxytocin-synthesizing neurons in the hypothalamus express PrRP receptors (26). PrRP activates oxytocin-synthesizing neurons and CRH-synthesizing neurons in the hypothalamus (27–29). Contextual conditioned fear activated the PrRP-synthesizing neurons in the medulla oblongata. In the MeA-lesioned rats, the percentage of PrRP-synthesizing neurons activated after contextual conditioned fear was significantly reduced. Furthermore, the neuroendocrine responses to contextual conditioned fear disappeared in PrRP-deficient mice. All of these findings suggest that the MeA-medullary PrRP-synthesizing neuron pathway is essential for neuroendocrine responses to contextual conditioned fear.

In the present study, the MeA lesions after training of contextual conditioned fear impaired neuroendocrine responses but not freezing behavior, suggesting selective involvement of the MeA in expression of neuroendocrine responses, although it remains to be determined whether the MeA is involved in controlling the peak amplitude or time course of neuroendocrine responses to contextual conditioned fear. It has been shown that the central amygdala lesion made after contextual fear conditioning blocks behavioral responses (18) but neither adrenocortical nor prolactin responses (19) (but also see Ref. 30) to contextual conditioned fear. The basolateral amygdala is the site of convergence of neural pathways that carry sensory information and the site of acquisition and storage of emotional memory (2, 3, 31). Neurons in the basolateral amygdala project directly or indirectly to the MeA (32). It is thus likely that the MeA, at least in part, mediates efferent signals from the basolateral amygdala to neuroendocrine cells. Consistent with this, the basolateral amygdala lesions made after conditioning blocked both neuroendocrine and behavioral responses to contextual conditioned fear (our unpublished observation) and cued conditioned fear (33).

The MeA lesions did not significantly change the magnitude of neuroendocrine responses to exposure to novel environmental stimuli. Because only 1 time point was examined in the present study, the present data do not completely exclude possible involvement of the MeA in response to novel environments. On the other hand, the responses to contextual conditioned fear were significantly impaired after the MeA lesions. Activation of oxytocin-synthesizing neurons after restraint stress has also been shown to be reduced after MeA lesioning (34). All of these findings suggest that involvement of the MeA in the control of neuroendocrine responses is dependent on the kind of stressful stimuli given.

Contextual conditioned fear activated the PrRP-synthesizing neurons in the NTS and VLM. The MeA lesion impaired activation of PrRP-synthesizing neurons in the NTS and VLM. However, direct projection of neurons in the MeA to the NTS or VLM was not confirmed in a previous anatomical tracing study (35). Neurons in the MeA send projections to the bed nucleus of the stria terminalis (BNST) and paraventricular nucleus (PVN) in the hypothalamus (35). Some of the neurons in the BNST and PVN directly project to the caudal NTS or VLM (36, 37). It is tempting to speculate that the BNST and/or PVN relay outputs of the MeA to the caudal NTS and VLM.

In the present experiments, PrRP-deficient mice did not exhibit neuroendocrine responses after contextual conditioned fear, although prolactin responses remain to be clarified. This finding is consistent with results of previous studies showing that post-training lesions of noradrenergic projections including PrRP fibers impaired activation of the hypothalamus and neuroendocrine responses after contextual conditioned fear and that immunoneutralization of endogenous PrRP with intracerebroventricular anti-PrRP antibodies impaired oxytocin secretion after contextual conditioned fear (10, 12, 16). Post-training MeA lesions impaired activation of PrRP-synthesizing neurons and neuroendocrine responses to contextual conditioned fear (Figures 1 and 3). All of these data suggest the importance of PrRP in expression of neuroendocrine responses to contextual conditioned fear, although further investigation is necessary because there were some differences in parameters of contextual fear conditioning between experiments for rats and mice (eg, way of exposing to contextual stimuli before conditioning, timing, and current intensity of electrical footshocks), and these differences might have induced different neural activation in rats and mice (38–40). The role of PrRP in acquisition of contextual conditioned fear remains to be determined. Medullary PrRP-synthesizing neurons project to the hypothalamus, which expresses PrRP receptors (16, 26, 41). Contextual conditioned fear activates PrRP-synthesizing neurons in the NTS and VLM that project to the SON (16). Oxytocin-synthesizing neurons in the SON and PVN express PrRP receptors, and PrRP facilitates oxytocin release from the isolated SON (26). In addition, it has been shown that CRH-synthesizing neurons in the PVN make contact with PrRP-positive fibers (42). All of these findings suggest that medullary PrRP-synthesizing neurons play a facilitative role in oxytocin and ACTH responses to contextual conditioned fear.

Medullary PrRP-synthesizing neurons contain noradrenaline (13–15). Neuroendocrine responses to contextual conditioned fear are impaired by administration of an α1-adrenoceptor antagonist (11) or disruption of noradrenergic fibers, some of which contain PrRP (10). Coadministration of PrRP and noradrenaline synergistically or additively induces ACTH release (41, 43), although it remains to be determined whether CRH-synthesizing neurons express both adrenoceptors and PrRP receptors. Precise roles of interactions between noradrenaline and PrRP in the control of neuroendocrine responses to contextual conditioned fear remain unclear.

Both MeA lesions and PrRP deficiency reduced or blocked neuroendocrine but not behavioral responses to contextual conditioned fear, suggesting differential roles of the MeA-medullary PrRP-synthesizing neuron pathway in the control of neuroendocrine and behavioral responses to contextual conditioned fear. On the other hand, the central amygdala-periaqueductal gray pathway has been shown to mediate freezing behavior in response to contextual conditioned fear (3, 17). Freezing behavior during contextual conditioned fear was significantly augmented in PrRP-deficient mice, suggesting an inhibitory role of PrRP in freezing behavior. Although sites of PrRP action to suppress freezing behavior remain to be clarified, it is interesting to speculate that PrRP influences the central amygdala-periaqueductal gray pathway.

In the present study, MeA lesions induced dissociation between behavioral and neuroendocrinologic responses to fear-related stimuli. Dissociation of behavioral and autonomic responses to a task load has been shown in subjects with posttraumatic stress disorder (44). Subjective feelings have been shown to be dissociated with autonomic/neuroendocrine responses to stressful stimuli in subjects with major depressive disorder (45) and with anorexia (46). Although physiological mechanisms underlying the dissociation remain to be clarified, it is interesting to speculate the possible involvement of the medial amygdala in pathogenesis of these diseases. The present study provides evidence that the MeA-medullary PrRP-synthesizing neuron pathway plays an important role in neuroendocrine responses to contextual conditioned fear. Clarification of neural fear circuitry is important for understanding neural bases of fear-related disorders, such as posttraumatic stress disorder, panic disorder, and phobia. Further investigation is needed to prove the clinical relevance of the MeA-medullary PrRP-synthesizing neuron pathway.
